# Protective Effect of SIRT1 Activator on the Knee With Osteoarthritis

**DOI:** 10.3389/fphys.2021.661852

**Published:** 2021-04-13

**Authors:** Zhenquan Zhou, Zhenhan Deng, Yuwei Liu, Yizi Zheng, Shiwei Yang, Wei Lu, Deming Xiao, Weimin Zhu

**Affiliations:** ^1^Department of Sports Medicine, The First Affiliated Hospital of Shenzhen University, Shenzhen Second People’s Hospital, Shenzhen, China; ^2^Department of Orthopaedics, Shenzhen Hospital of Southern Medical University, Shenzhen, China; ^3^Clinical Medical College, Guangzhou Medical University, Guangzhou, China; ^4^Clinical Medical College, Shenzhen University, Shenzhen, China; ^5^Clinical Medical College, Guangxi University of Chinese Medicine, Nanning, China; ^6^Clinical Medical College, Anhui Medical University, Hefei, China; ^7^Department of Thyroid and Breast Surgery, Shenzhen Breast Tumor Research Center for Diagnosis and Treatment, National Standardization Center for Breast Cancer Diagnosis and Treatment, Shenzhen Second People’s Hospital, The First Affiliated Hospital of Shenzhen University, Shenzhen, China; ^8^Teaching Office, Shenzhen Second People’s Hospital, Shenzhen, China

**Keywords:** osteoarthritis, resveratrol, SIRT1, p53, cartilage, Micro-CT

## Abstract

Osteoarthritis (OA), one of the most common chronic musculoskeletal disorders, is deemed to be correlated with aging. The SIRT1 activator, resveratrol, acts as a crucial regulator of aging and may have a potential therapeutic effect on OA. Rabbit OA models were established through destabilized medial meniscus surgery. A total of 40 healthy male New Zealand rabbits were divided into five groups: control group (sham operation), OA group, as well as low dose (LD), middle dose (MD), and high dose (HD) resveratrol-treated OA groups. 6 weeks after operation, 0.8 ml of normal saline was injected into the knee joints every other day in the control and OA groups, and 0.8 ml of 5, 10, and 15 μmol/L resveratrol was injected into the knee joints every other day in the LD, MD, and HD group, respectively. The rabbits were sacrificed 2 weeks after medication, and the articular cartilage of the knee joint was collected for Micro-CT, histology and Western blot analysis. Obvious articular cartilage lesion and joint space narrowing were detected in the OA group. Compared with the OA group, less osteoarthritic changes were observed in the MD and HD groups. The MD and HD groups had significantly lower bone volume fraction, trabecular number and Mankin scores than the LD and OA groups (*p* < 0.05). No significant difference was found between the OA and LD groups (*p* > 0.05). The expressions of SIRT1 and p53 detected by western blot were consistent with the aforementioned findings. Therefore, resveratrol can activate the SIRT1 gene to play a protective role in the OA process by inhibiting chondrocyte apoptosis, trabecular bone number increasing of the subchondral bone, as well as elevation of bone density. It demonstrated the importance of SIRT1 in maintaining articular cartilage health and provided a promising therapeutic intervention in the treatment of OA.

## Introduction

Osteoarthritis (OA) is one of the most common chronic musculoskeletal disorders (Glyn-Jones et al., 2015; Deng et al., 2018). There are multiple treatments in clinical practice, but none of them can inhibit the pathological processes effectively. Arthroplasty is an effective treatment for end-stage OA; however, in view of the serious complications of surgery and limited lifespan of prostheses, researchers are pursuing treatments for early OA and measures for disease prevention (Glyn-Jones et al., 2015). The epidemiology of OA is complex and multifactorial, and has not yet been fully understood, so how to treat OA is still a great challenge worldwide. Among various risk factors, age-related changes in the phenotype of articular chondrocytes are key factors in the development and progression of OA (Rahmati et al., 2017). The mechanism of aging in relation to OA, which is still unclear, could provide targets for therapy in OA treatments.

Sirtuins are members of the class III histone deacetylase family, which mainly regulate diverse cellular activities in aging and aging-related diseases (Deng et al., 2019b). The silent information regulator 2 type 1 (also known as sirtuin 1 [SIRT 1]) is the most popular sirtuin homologs and regulates many vital signaling pathways such as DNA repair and apoptosis, myogenic and adipogenic differentiation, mitochondrial biogenesis, and glucose and insulin homeostasis (Hubbard and Sinclair, 2014). Resveratrol (Res, 3, 5, and 4’-trihydroxy-*trans*-stilbene), a non-flavonoid polyphenol compound, can activate the SIRT1 gene (Deng et al., 2019b). Studies have examined its anti-inflammatory, antioxidant, anti-free radical, anticancer effects as well as the function of improving micro-circulation (Ota et al., 2006; Moussa et al., 2017; Xia et al., 2017). Although evidence has shown that the SIRT1 gene plays an important role in chondrocyte apoptosis, but the specific mechanism is still unclear (Takayama et al., 2009). A study on the other cell line indicated that the equilibrium state of nuclear factor-kappaB (NF-κB) and p53 regulated by the SIRT1 gene had a significant impact on cell survival (Day et al., 2001). A previous study also revealed that the forkhead box O (FOXOs) gene regulated chondrocyte apoptosis by modulating the expressions of apoptosis-related factors (Goldring and Goldring, 2010). The interaction between SIRT1 and FOXOs improves cell survival (Shane Anderson and Loeser, 2010), and provides a basis for investigating the role of SIRT1 in regulating OA progression.

As a type of gene closely related to cell differentiation, aging and apoptosis, the role that the SIRT1 gene plays in the occurrence and development of OA is still unclarified. In this study, we supposed that the SIRT1 gene participated in chondrocyte aging and apoptosis by modulating the expressions of NF-κB and p53. Meanwhile, the therapeutic effect of resveratrol in rabbit OA models was explored.

## Materials and Methods

### Ethics Statement

The animal experiment was carried out in accordance with relevant guidelines and regulations, and was approved by the Medical Ethics Committee of the First Affiliated Hospital of Shenzhen University, Shenzhen Second People’s Hospital.

### Animals and Grouping

A total of 40 3-month old male mature New Zealand white rabbits (1.8 ± 0.17 kg) provided by Guangdong Medical Laboratory Animal Center were used in this study. Each of the rabbits was housed in an individual cage under the following conditions: 21 ± 1°C; 12 h/12 h light/dark cycle; 40–60% relative humidity; and fed with water, rabbit chow, and green leaves.

All the rabbits were randomly divided into five groups (eight in each group): control group (sham operation), OA group (OA induction with normal saline injection), LD group (low dose resveratrol, OA induction with 5 μmol/L of resveratrol injection), MD group (moderate dose resveratrol, OA induction with 10 μmol/L of resveratrol injection), and HD group (high dose resveratrol, OA induction with 15 μmol/L of resveratrol injection).

### Destabilized Medial Meniscus – Induced OA Model

All surgeries were performed under isoflurane anesthesia, and the rabbits were given postsurgical buprenorphine (0.05 mg/kg subcutaneously) as analgesia.

According to the reference, destabilized medial meniscus (DMM) surgery was performed randomly on one side of the lower leg to create OA models (Deng et al., 2019a). Briefly, the rabbit was fixed on the operation table with the right leg in 90° flexion. The joint capsule was opened with an incision just medial to the patellar tendon, and the medial meniscotibial ligament was sectioned. Then, meniscus was removed to eventually achieve the OA model. Sterility requirements were followed during operation. Joints were wrapped by iodine gauze and fixed with elastic bandage. Normal feeding was performed after operation for 2 weeks. In the first 3 days postoperatively, 800,000 units of penicillin (Shijiazhuang Pharmaceutical Group, China) were intramuscularly injected on a daily basis to prevent infection.

Six weeks after operation, 0.8 ml of normal saline was injected into the articular cavity of the surgery knee every other day in the control group, and 0.8 ml of resveratrol at the concentration of 5, 10, and 15 μmol/L (Shenzhen Xinhailing Biotechnology Co., Ltd.) was injected into the knee joints every other day in the LD, MD, and HD group, respectively.

### Sample Preparation and Cell Culture

Rabbits were sacrificed 8 weeks after operation. Biopsies of the cartilage and bone were acquired from the tibia plateau, including the loading zone and the margin zone as far as possible (Deng et al., 2017). Specimens were examined by Micro-CT. Articular cartilage was removed from the articular surface by sterile scalpel and was transferred to 4°C sterile DMEM (Hyclone, United States). After washing with phosphate buffer saline, it was then moved to a 150 ml flask containing 0.25% trypsin-ethylene diamine tetraacetic acid (EDTA, Gibico, United States). Samples were stirred and incubated for 1 h at 37°C and 95% humidity. Liquid was discarded and 0.02% collagenase II with 5% calf serum was added. Subsequently, samples were stirred and incubated overnight, and were filtered and centrifuged on the next day. Precipitates of chondrocytes were incubated with unfiltered cartilage. Culture medium was replaced every 3 days. Subculture was performed when chondrocytes covered 80% of the surface of culture medium.

### X-Ray Observation

Full leg X-rays of rabbits were captured using a Direct Radiography image system (SHIMADZU, Japan, 400 mA; Radiology Department, Shenzhen Second People’s Hospital). Kellgren-Lawrence (KL) classification score (0–4) was used to evaluate disease severity. A higher score indicates a higher level of OA severity.

### Micro-CT Analysis

The proximal tibia was scanned with a Scanco Viva CT 40 (Scanco Medical, Wangen-Brüttisellen, Switzerland) at 15-mm resolution 8 weeks after surgery. The X-ray energy was 100 mA and 50 kVp. The slice thickness was 15 mm. After obtaining 2-dimensional image slices, the field of interest was uniformly delineated, and three-dimensional (3D) reconstructions were performed with gauss = 0.8, s = 1, and threshold = 163 throughout the analysis. The bone volume/tissue volume ratio (BV/TV), trabecular number (Tb.N), trabecular thickness (Tb.Th), and the changes of 3D structure were analyzed through Micro-CT scanning.

### Histology

Dissected joints were fixed overnight at 4°C in 4% paraformaldehyde, decalcified for 3 weeks in 0.5 M EDTA pH = 7.5, embedded in paraffin, and then sectioned at 5 μm thickness in the sagittal plane. The sections were stained with hematoxylin and eosin (H&E; Deng et al., 2019a). The morphology of the articular cartilage was further evaluated using the Mankin scoring system. A higher score indicates a higher level of severity of cartilage degeneration.

### Western Blot

The second generation of chondrocytes cultured was used to test the signal pathway involved in resveratrol treatment. Chondrocytes were lysed with the RIPA buffer to extract the whole proteins. The total protein content of the extracted sample was quantified with a bicinchoninic acid protein assay. Protein samples (50 μg) were separated on 10% polyacrylamide gel and then transferred to polyvinylidenefluoride membranes. After blocked with 5% Skim milk/BSA, the membranes were incubated with primary antibodies including: GAPDH (1:1000, #5174, Cell Signaling Technology, Danvers, MA, United States), SIRT1 (#p475, 1:1000, Cell Signaling Technology, Danvers, MA, United States), and p53 (1:1000, #2527, Cell Signaling Technology, Danvers, MA, United States) overnight. After washing, the membranes were then incubated with secondary antibody (1:5000, #7074; Cell Signaling Technology, Danvers, MA, United States) for 2 h. Finally, the protein was exposed with diaminobenzidine. All experiments were repeated for three times.

### Statistical Analysis

The values were expressed as means ± standard deviation (SD). All statistical analyses were performed using the SPSS 16.0 software (Chicago, IL, United States). One-way ANOVA followed by LSD’s *post hoc* tests was used to determine the differences among groups. Student’s *t* test was used to compare between two groups. *P* value < 0.05 was considered statistically significant.

## Results

### X-Ray Observation

**FIGURE 1:**
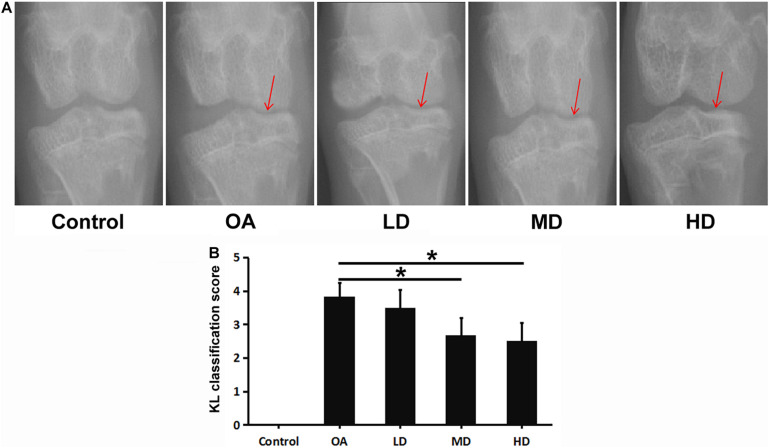
X-ray of the knee in each group at 8 weeks after operation. Arrows showed the joint space narrowing and articular cartilage lesion of the knee joint. **(A)** X-ray of the knee in each group. **(B)** KL classification score of the knee in each group. *P < 0.05.

In the control group, no significant change of the joint space was found, and smooth and clear outline of the articular surface was observed both in femoral condyle and tibial plateau. In the OA group, narrowed medial space of knee joint and rough surface of tibial plateau was observed. For the resveratrol treatment groups with different doses, the medial space of knee joint gradually narrowed relative to the control group as the dose decreased, but was wider than that of the OA group. The articular surface of femoral condyle and tibial plateau in the LD, MD, and HD groups appeared to be smoother compared to the OA group (Figure 1A).

The OA, LD, MD, and HD groups had higher KL classification scores than the control group (*P* < 0.05). The KL classification scores in the MD and HD groups were significantly lower than those in the OA and LD groups (*P* < 0.05), while no significant difference was found between the MD and HD groups, and between the OA and LD groups (*P* > 0.05, Figure 1B).

### Micro-CT Analysis

**FIGURE 2:**
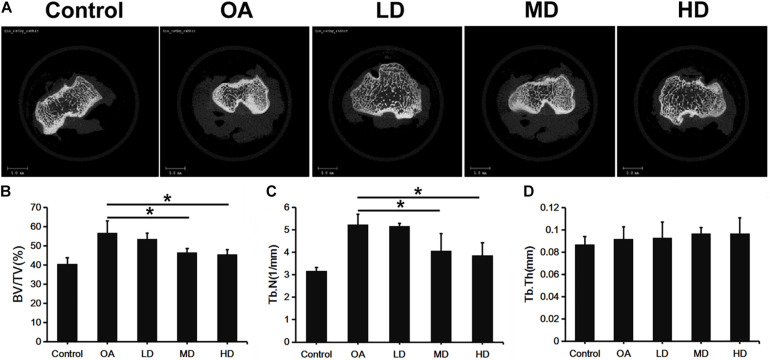
Micro-CT analysis of proximal tibia of difference groups at 8 weeks after surgery. **(A)** Representative Micro-CT 3D images showed bone microarchitecture of proximal tibia of different groups. Scale bar = 5 mm. **(B–D)** Quantification of BV/TV, Tb.N, and Tb.Th of different groups. *P < 0.05.

Figure 2A presents the 3D images of proximal tibial. The OA, LD, MD, and HD groups had higher BV/TV values than the control group (*P* < 0.05). The BV/TV values in the MD and HD groups were lower than those in the control, OA and LD groups (*P* < 0.05), while no significant difference was found between the MD and HD groups, and between the control and LD groups (*P* > 0.05, Figure 2B).

The Tb.N value showed a consistent trend with BV/TV. The OA, LD, MD, and HD groups had lower Tb.N values than the control group (*P* < 0.05). The Tb.N values in the MD and HD groups were lower than those in the control, OA and LD groups (*P* < 0.05), while no significant difference was found between the MD and HD groups, and between the control and LD groups (*P* > 0.05, Figure 2C).

No significant difference in the Tb.Th value was detected among all the groups (*p* > 0.05, Figure 2D).

### Histological Analysis

**FIGURE 3:**
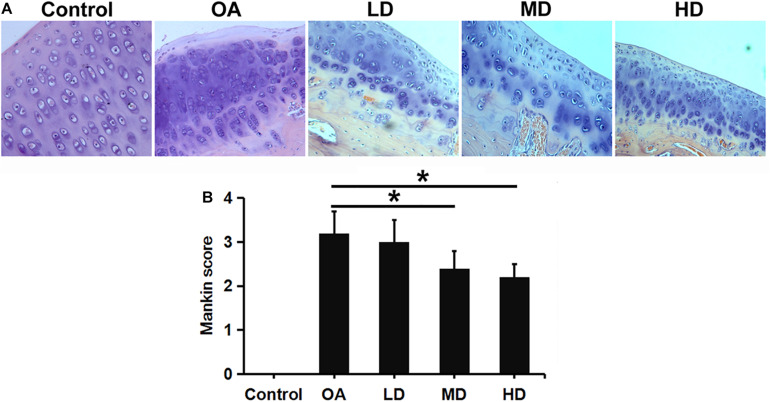
H&E staining and Mankin score evaluation of articular cartilage of the knee in different groups. **(A)** H&E staining showed articular cartilage of the knee in different groups (200X). **(B)** Mankin score evaluation of different groups. *P < 0.05.

H&E staining showed that chondrocytes were orderly arranged in the control group, with the shallow layer, transitional layer, radiation layer, and calcified layer presented from top to bottom in columnar array. Chondrocyte clusters were not observed and the tidemark was intact. In the OA group, the cartilage surface was rough, cracks were seen, and the chondrocyte number was decreased and disorderly arranged and clustered. The morphology of the cartilage was gradually improved with the increase of resveratrol dose (Figure 3A).

Mankin score for OA evaluation was adopted in this study. A higher score indicates greater cartilage lesion. As the dose of resveratrol increased, The resveratrol treatment groups presented gradually-decreasing scores compared to the control and OA groups. The MD and HD groups showed significantly lower Mankin scores than the LD and OA groups (*P* < 0.05). No difference was found between the MD and HD groups, and between the LD and OA groups (*P* > 0.05, Figure 3B).

### Western Blot Analysis

**FIGURE 4:**
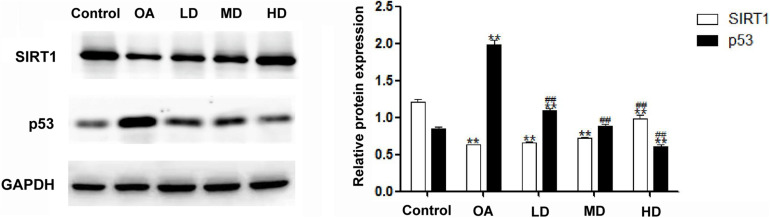
Expressions of SIRT1 and p53 detected by Western blot. **P < 0.05, compared with the control group; ^##^*P* < 0.05, compared with OA group.

The Western blot assay showed that the expression of SIRT1 in the control group was higher than that in other groups (*P* < 0.05), and the SIRT1 expression in the MD and HD groups was higher than that in the OA and LD groups (*P* < 0.05). The expression of p53 in the OA group was higher than that in other groups (*P* < 0.05), and p53 expression in the control group was lower than LD group (*P* < 0.05), higher than MD group (*P* < 0.05), and no difference when compared with MD group (*P* > 0.05, Figure 4).

## Discussion

In this study, it was found that the SIRT1 activator, resveratrol, inhibited the process of articular cartilage lesion and provided protection against OA. We revealed a signaling model in cartilage linking the p53 signal pathway with the SIRT1 activator, resveratrol.

The loss of cartilage in subchondral bone is a typical pathological change and feature in OA. In the later stage of OA, the pathological changes of subchondral bone include eburnation, bone cyst, and osteophyte (Chen et al., 2020). We compared BV/TV, Tb.N, and Tb.Th between different groups through Micro-CT scanning. Compared with the LD and OA groups, the HD group exhibited a stronger inhibiting effect on the increase of Tb.N and density, thus retarding the formation of eburnation, bone cyst and osteophyte. Among the various factors, aging of cartilage is an important one contributing to the development of OA. It also exerts a synergism effect along with other OA risk factors such as inflammation, oxidative stress, and changes in the extracellular matrix (Loeser et al., 2016). The reduction of muscle and accumulation of fat will change the joint loading and lead to adipokine and cytokine production and systemic inflammation, which will give impetus to OA (Greene and Loeser, 2015). The excessive reactive oxygen species is correlated with age associated oxidative stress and will result in aging diseases via cellular damage (Jones, 2015). The accumulation of advanced glycation end-products, which will lead to non-enzymatic collagen cross-linking, can change the mechanical properties of the extracellular matrix of cartilage, thus resulting in cartilage degeneration (Verzijl et al., 2002).

It was suggested that resveratrol, a polyphenol found in grapes, could act as an activator of SIRT1 to protect against oxidative damage, inflammation, and cancer (Lee et al., 2019). In our study, resveratrol exhibited the ability to modulate Tb.N and density. Observation was performed under optical microscope after morphology staining. In the control group, chondrocytes appeared to be normal and orderly arranged, with the shallow layer, transitional layer, radiation layer, and calcified layer presented in columnar array. Chondrocyte clusters were unseen, and the tidal line was intact. The changes in chondrocytes differed among the experimental groups, which accorded with pathological changes in OA. The Mankins score also supported the protective effect of resveratrol with regard to cartilage lesion. Resveratrol can regulate multiple molecular targets to increase DNA stability and hinder the process of aging-related diseases (Deng et al., 2019b). It also showed the positive effects of calorie restriction and lifespan extension (Deng et al., 2019b). Meanwhile, resveratrol can inhibit reticulum stress (ER stress) and prevent cell death via autophagy pathways (Lee et al., 2019).

The results of the expressions of SIRT1 and p53 detected by Western blot indicated that resveratrol at different doses could alleviate pathological changes in the OA model. In the MD and HD groups, resveratrol showed a better protective effect than in the LD group, implying that the effect of resveratrol may be concentration dependent. It was further confirmed that resveratrol played an important role in chondrocyte apoptosis by activating SIRT1. Previous studies had confirmed that the expression of SIRT1 decreased in seriously degenerated cartilage (Fujita et al., 2011). Our results showed the same trend: the control group had the highest while the OA group had the lowest SIRT1 expression, and the SIRT1 expression increased with the increase of resveratrol dose. The catabolic, oxidative stress and the pro-inflammatory factors reduced the SIRT1 expression and activity in articular chondrocytes (Deng et al., 2019b). SIRT1 has been suggested to play a vital role in several OA processes. The increased level of SIRT1 is correlated with up-regulated collagen II and aggrecan expression (Oppenheimer et al., 2014). SIRT1 is essential in chondrogenic differentiation of mesenchymal stem cells via NF-κB inhibition and SOX9 activation (Buhrmann et al., 2014). The opposing effects of SIRT1 with matrix metalloproteinases enzymes and tumor necrosis factor-α on cartilage showed the anti-catabolic and anti-inflammatory effects of SIRT1 (Deng et al., 2019b). The anti-oxidative stress effects of SIRT1 were also confirmed through its upregulation after oxidant insult and its decreasing under melatonin treatment in chondrocytes (Lim et al., 2012). The p53 protein is one of the first deacetylated non-histone proteins that are proved to interact with SIRT1. Under cellular stress, p53 is induced by phosphorylation and acetylation and is significantly up-regulated (Tang et al., 2008). Thus, the deacetylation by SIRT1 modulates the function of p53. In this study, along with the alleviation of OA severity, SIRT1 was increased. Consequently, p53 was dramatically decreased in the groups treated with resveratrol, indicating reduction of apoptosis in OA tissue.

Animal models of OA are used worldwide to address the disease mechanism and to investigate prevention methods and treatments. As rabbits share a similar structure of knee with human, they can be utilized to help us discover the pathological processes, features, and metabolism of OA. Thus, the choice of DMM-OA model in our study is feasible. This method is worldwide recognized and adopted. A successful animal model is the key to the present study.

In our study, it was confirmed that the SIRT1/p53 signal pathway played an important role in aging and apoptosis in chondrocytes (Deng et al., 2019b). A previous study also reported the anti-apoptosis and autophagy effect of SIRT1 by activating the insulin-like growth factor, the phosphatidylinositol 3-kinase signal, and the mammalian target of rapamycin pathway (Gagarina et al., 2010). Above all, our study provided evidence on the protective effect of resveratrol in articular cartilage via the SIRT1/p53 signal pathway. In view of the low bioavailability of resveratrol, further investigation is needed to improve its bioavailability and therapeutic effects in clinical trials.

## Conclusion

Resveratrol can activate the SIRT1 gene to play a protective role in the OA process by inhibiting chondrocyte apoptosis, trabecular bone number increasing of the subchondral bone, and elevation of bone density. It demonstrated the importance of SIRT1 in maintaining articular cartilage health and provided a promising therapeutic intervention in the treatment of OA.

## Data Availability Statement

The original contributions presented in the study are included in the article/supplementary material, further inquiries can be directed to the corresponding author/s.

## Ethics Statement

The animal study was reviewed and approved by Medical Ethics Committee of The First Affiliated Hospital of Shenzhen University, Shenzhen Second People’s Hospital.

## Author Contributions

DX and WZ designed and directed the study. ZZ, ZD, and YL performed the experiments. YZ and ZD analyzed the data. SY and WL collected the data. ZD and YL prepared the manuscript. ZD and WZ were responsible for funding acquisition. All authors contributed to the article and approved the submitted version.

## Conflict of Interest

The authors declare that the research was conducted in the absence of any commercial or financial relationships that could be construed as a potential conflict of interest.

## Abbreviations

3D: Three-dimentional
AGEs: Advanced glycation end-products
BCA: Bicinchoninic acid
BV/TV: Bone volume/tissue volume ratio
DAB: Diaminobenzidine
DMM: Destabilized medial meniscus
EDTA: Ethylene diamine tetraacetic acid
FOXOs: Forkhead box O
H&E: Hematoxylin and eosin
HD: High dose
IGF: Insulin-like growth factor
KL: Kellgren-Lawrence
LD: Low dose
MD: Middle dose
MMPs: Metalloproteinases
mTOR: Mammalian target of rapamycin
NF- κ B: Nuclear factor-kappaB
OA: Osteoarthritis
PBS: Phosphate buffer saline
PI3K: Phosphatidylinositol 3-kinase
ROS: Reactive oxygen species
SD: Standard deviation
SIRT 1: Sirtuin 1
Tb.N: Trabecular number
TNF- α: Tumor necrosis factor- α
Tb.Th: Trabecular thickness.
